# Does the Wage Gap between Private and Public Sectors Encourage Political Corruption?

**DOI:** 10.1371/journal.pone.0141211

**Published:** 2015-10-23

**Authors:** Boris Podobnik, Vuk Vukovic, H. Eugene Stanley

**Affiliations:** 1 Center for Polymer Studies and Department of Physics, Boston University, Boston, MA 02215, United States of America; 2 Faculty of Civil Engineering, University of Rijeka, 51000 Rijeka, Croatia; 3 Faculty of Economics, University of Ljubljana, 1000 Ljubljana, Slovenia; 4 Zagreb School of Economics and Management, 10000 Zagreb, Croatia; 5 Adriatic Economic Association, 10000 Zagreb, Croatia; 6 Luxembourg School of Business, Luxembourg; 7 Adam Smith Institute, 23 Great Smith Street, London, United Kingdom; University of Maribor, SLOVENIA

## Abstract

We present a dynamic network model of corrupt and noncorrupt employees representing two states in the public and private sector. Corrupt employees are more connected to one another and are less willing to change their attitudes regarding corruption than noncorrupt employees. This behavior enables them to prevail and become the majority in the workforce through a first-order phase transition even though they initially represented a minority. In the model, democracy—understood as the principle of majority rule—does not create corruption, but it serves as a mechanism that preserves corruption in the long run. The motivation for our network model is a paradox that exists on the labor market. Although economic theory indicates that higher risk investments should lead to larger rewards, in many developed and developing countries workers in lower-risk public sector jobs are paid more than workers in higher-risk private sector jobs. To determine the long-run sustainability of this economic paradox, we study data from 28 EU countries and find that the public sector wage premium increases with the level of corruption.

## Introduction

Political corruption is arguably one of the main factors constraining wealth creation and economic growth in modern democracies [1–11]. It can be defined as systemic misuse of primary government institutions—laws and regulations are altered to offer private benefits to politicians and government officials [5, 7]. Because an increase in corruption and state interference increases the motivation to seek political patronage and clientelism, a highly corrupt system changes incentives of market participants. They get skewed towards gaining political favors instead of competing in the marketplace. In countries more prone to corruption this implies an enormous level of inefficiency. Highly corrupt countries tend to be poorer and are usually developing or transitional economies, often of a socialist origin [11]. They also tend to be less open, have a higher regulation of market entry, lower freedom of the press, and steadily depleting human capital [1, 8–11].

To quantify incentives for engaging in corruption that causes such distorted market outcomes, we evaluate the relative riskiness of working in the public vs. private sector. Although we would assume that the level of risk corresponds to job safety and that there should be a higher wage premium for a riskier position, the data gathered on a sample of 28 EU countries indicates that the lower-risk public sector job tends to hold a higher wage premium, i.e. the remuneration for a public sector job, which is safer and offers greater benefits, is higher than for a corresponding private sector job. Even in the United States, according to the Congressional Budget Office [12], jobs in the public sector carry greater advantages than jobs in the private sector. They are more secure, less stressful, offer a wider selection of health-insurance plans, better retirement benefits, flexible work arrangements, and more holiday and vacation days per year. The question is: how is this discrepancy, in which the less riskier sector is rewarded with higher wages, sustainable in the long run?

Our hypothesis is that in countries with high public sector wage premiums the probability that political corruption will be present is higher. Intuitively, the greater the gap the higher the probability that public sector jobs are being allocated not based on merit but on political connections, nepotism, and bribery [13]. Because politicians in corrupt countries shun accountability towards their voters, they can increase the levels of patronage and nepotism. Thus the greater the public sector wage premium, the greater the motivation for those without adequate skills and education to enter the public sector and achieve private benefits. The wage discrepancy is in obvious contrast to economic theory that higher risk must be followed by greater reward. To quantify how well a given wage compensates a worker for the risk incurred, we propose a new labor reward-to-risk ratio.

Finally, using the same logic that explains why the wage premium tends to be high in more corrupt countries, we model how the better connected corrupt minority makes corruption grow and persist in democracies. We present a dynamic network model of corrupt and noncorrupt agents representing two states in public and private sectors in a corrupt environment. Corrupt agents are more connected to one another and are less willing to change their attitudes regarding corruption than noncorrupt agents. This behavior enables them to prevail and become the majority in the workforce through a first-order phase transition. In our simple model democracy thus serves as a mechanism of preserving corruption in more corrupt countries, the same way it prevents corruption in low-corrupt countries.

## Data

Using data from a European Commission report [14] and the International Labor Organization (ILO) [15] we analyze employee wage levels in the public and private sectors in 28 EU countries. Using the ILO LABORSTA database, we examine wages across similar occupations in both the public and private sector in 75 countries worldwide. Public sector wages (those associated with public administration, schools, and medical facilities) are calculated based on a 10-year average, from 1999 to 2008. Private sector wages (i.e., all other listed occupations excluding agriculture) are also calculated based on the same 10-year average. Although some jobs in one sector do not have an exact counterpart in the other, we find that the jobs in the two sectors are sufficiently similar that we can make useful comparisons. We compare similar job positions across both sectors (e.g., cleaning personnel, bus drivers, electricians, engineers, managers, IT personnel, administration officers, and secretaries). Obviously the gap in wages varies with different levels of education, however in the final calculation we take the averages across all occupations to calculate the gap. The data from the ILO database gives us the same prediction as the data from the EU wage gap report [14].

To measure corruption we use the Corruption Perception Index (CPI) supplied by Transparency International [16] from 1999 to 2013. The CPI is a composite index based on independent surveys of individuals and businesses provided by more than ten independent world-wide institutions. The index measures public perceptions of abuse by officials, extent of media control, level of accountability, persistence of bribes, and level of judiciary independence. The CPI values range from 0 (highly corrupt) to 100 (highly transparent). Thus the larger a country’s CPI, the less corrupt the country. We also gather data on GDP growth and GDP per capita in 2011 PPP terms from the World Bank database from 1999 to 2013 [17].

## Results

### Evolution of the wage gap

**Fig 1:**
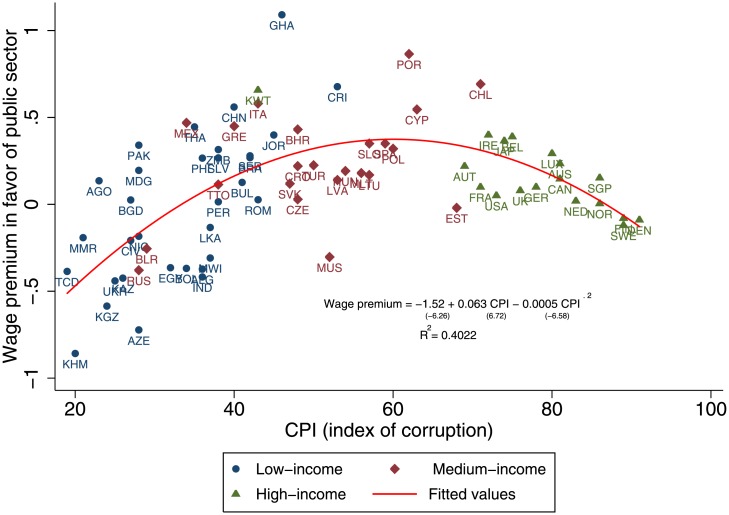
Evolution of the wage gap with the level of development. In this and all other figures, the regression line t-values for the corresponding coefficients are shown in parentheses, and standard errors are robust to heteroskedasticity. The income separators are $15,000 and $35,000 of PPP adjusted GDP per capita.

Previous studies [11] suggest that the negative connection between corruption level and country wealth takes the form of a power-law functional dependence [18]. Our hypothesis here is that across different stages of development when the wage gap between the two sectors is skewed in favor of the public sector, the motivation to engage in corruption is increased. We observe this relationship (Fig 1) using the set of 75 countries (ranging from undeveloped to developed) and find an inverted U-shaped functional dependence between the public sector wage premium and the CPI-quantified corruption level (the *t*-statistic of CPI^2^ is −6.58) with clear clustering exhibited among the three major income groups: low-income, middle-income, and high-income countries.

Fig 1 shows that in most low-income countries (blue circles) public sector workers are relatively underpaid compared to their private sector counterparts, as the state fails to perform its basic functions, not being able to afford higher public wages thus making public institutions weak. This suggests that these underpaid public sector workers will be more motivated to compensate for their low wages through corruption and taking bribes. The standard literature on corruption and wages [19] supports this argument. In this set of countries public sector corruption tends to be individualized, not systemic. Fig 1 also shows that in most medium-income countries (dark red diamonds) corruption can grow if there is a steadily increasing wage imbalance that over-inflates public-sector wages and disrupts incentives for work. Finally, Fig 1 shows that in most high-income countries (green triangles) the wage gap is greatly reduced and corruption is low.

### Wage gap, corruption and GDP growth for EU countries

**Fig 2:**
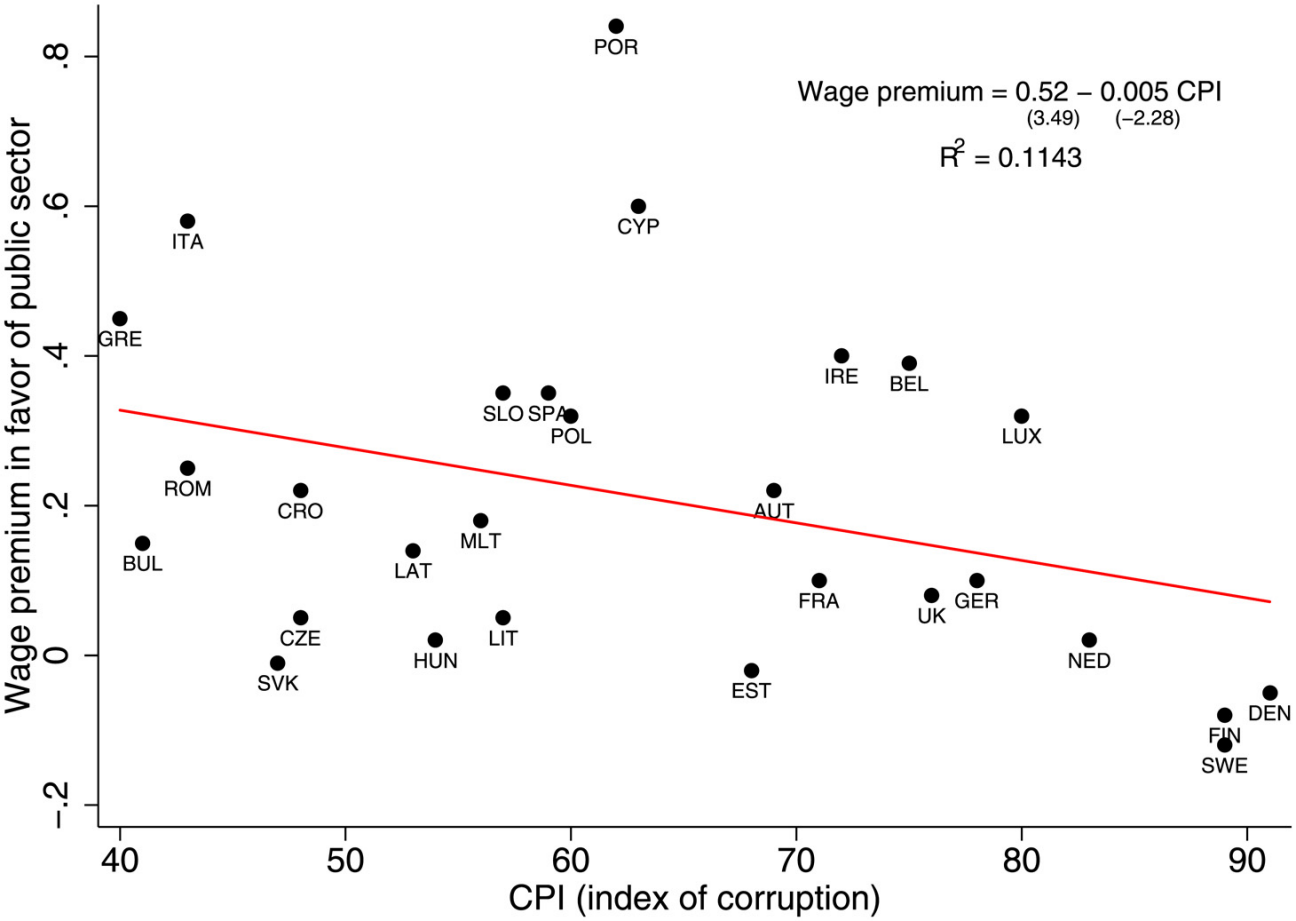
EU countries: The higher the corruption (lower CPI) the higher the public sector wage premium.

Because it is often difficult to analyze a heterogeneous group of countries ranging from low-income to high-income, as in Fig 1, we analyze a more homogeneous group of 28 EU countries that excludes the low-income countries shown in the left section of the parabola in Fig 1. Fig 2 shows the wage gap between the 10-year average wage in the public sector and the 10-year average wage in the private sector and compares it with the 2013 corruption level. We focus on the sample of institutionally stable EU countries because some of the public sector jobs in the EU (e.g., health, education, and science) tend to be held by individuals that are better educated than their counterparts in non-EU countries—particularly those in the developing world—and thus any comparison could be biased. Recalling that a larger CPI implies lower corruption, Fig 2 indicates that the more corrupt a country is, the greater will be the wage gap between its public and private sectors. We fit a functional dependence in Fig 2 with a power law with *α* = −0.005, a *t*-statistic of −2.28, and an adjusted *R* ^2^ of 0.11.

**Fig 3:**
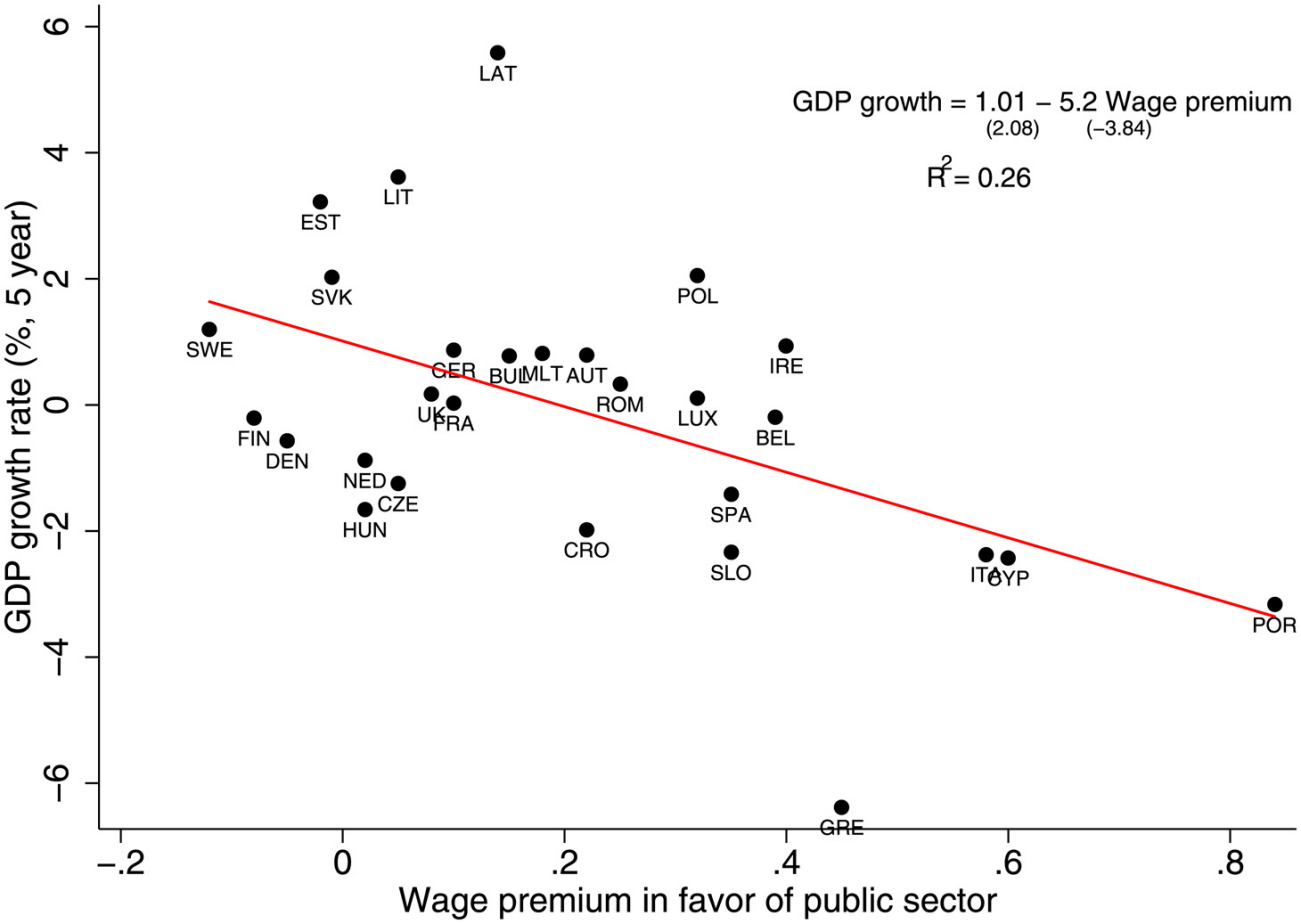
The smaller the wage gap between public and private sectors in favor of the public sector, the larger the average 5-year GDP growth rate.

Finally, to show how a public sector wage premium affects a country’s wealth, Fig 3 presents a clear negative correlation between the average five-year GDP growth rate and the wage premium for a set of 28 EU countries (*α* = −5.2, *t*-statistic = −3.84, *R* ^2^ = 0.26). The given correlations say nothing about the causal relationship between corruption and the wage gap. They serve as a mere motivation for our network model in which we aim to uncover why would a country with a higher public sector wage premium have higher levels of corruption.

## Wage Gap as a Motivation for Corruption

### Income to Risk as Reward-to-Risk Ratio in Labor Economics

In finance it is widely accepted that bonds are less risky than stocks and thus, on average, bonds should bring smaller returns than stocks. Similarly, if public sector jobs are less risky than private sector jobs, wages in the public sector should be smaller than wages in the private sector. This is frequently not the case. We speculate that this is because the corrupt minority within the public sector uses its powerful influence to preserve the system from which they are benefiting.

In corrupt countries in which the reward for membership in the dominant political party is a higher-wage public sector job, public sector jobs tend to become packed with under-qualified individuals who are prized for their obedience rather than for their ability to carry out the work [13]. In this scenario, gaining political favors (public sector jobs) as a party member is easier than competing in the marketplace. This disturbs incentives for work in both sectors and creates a strong motivation for those in the better paid, low-risk sector to protect their positions.

Although there are many possible definitions of risk in labor economics, we define it in terms of the average time a worker is able to continue in his or her job. Using this definition we immediately see that public sector jobs are more stable [20], i.e., on average they last longer than private sector jobs. This difference in risk level becomes particularly obvious during recessions and economic downturns when many more private sector jobs disappear than public sector jobs.

If we assume that holding a job in the private sector is riskier than holding one in the public sector, the next goal is to quantify this difference. We do this by measuring the average time agents in both sectors are able to work during their entire business career. We assume that the average business time in the private sector, $\tau_{\text{pr}}$, is smaller than the average business time in the public sector, $\tau_{\text{pu}}$. We define the risk in a given sector as the reciprocal average business time. Thus the risk of a public sector is $1/\tau_{\text{pu}}$, and the risk of a private sector is $1/\tau_{\text{pr}}$. If every job is equally available to every agent—usually not the case in a corrupt country—we start with the assumption that two agents (two engineers or two bus drivers) with equal skills and education in both sectors should make the same income during their entire business career. Thus we assume the *average annual income* (S) to be

$$\begin{array}{c} S_{\text{pr}}\;\tau_{\text{pr}}=S_{\text{pu}}\;\tau_{\text{pu}}. \end{array}$$ (1)

Here the total income made by each agent should be the same for both sectors. The parameter S*τ* can be further expressed as the ratio

$$\begin{array}{c} \frac{S}{\sigma }\equiv \frac{S}{1/\tau }, \end{array}$$ (2)

which characterizes how much income (i.e., the reward) compensates employees for risk (*σ*) in a given sector. Eq (1) gives the relative compensation between the two sectors.

As stated above, the ratio shown in Eq (2) utilizes the reward-to-risk ratio defined in finance, where agents compare investment opportunities by examining both the possible returns and the risk levels involved in each investment [21]. The labor economics reward-to-risk ratio shown in Eq (2) refers to the relationship between the potential reward from a given sector and the risk present in the same sector. As stated above, a public sector job generally offers more privileges and benefits than a private sector job (e.g., greater health care coverage). For that reason the risk in the private sector job is somewhat greater than that expressed in Eq (2). Taking into account the additional privileges granted in public sector jobs *ϕ* (and the expenses incurred in private sector jobs), we assume that workers in the private sector should on average make more money during their entire business careers than workers in the public sector. Thus we assume the *average annual income* (S) to be $S_{\text{pu}}\;\tau_{\text{pu}}=\phi \;S_{\text{pr}}\;\tau_{\text{p}}$, where *ϕ* < 1. This new parameter *ϕ* increases the average annual income of the private sector and additionally increases the risk of the private sector

$$\begin{array}{c} \sigma_{\text{pr}}\equiv \frac{1}{\phi \;\tau_{\text{pr}}}=\frac{1}{\tau_{\text{pr}}}+\sigma_{\text{pr}}^{\prime }. \end{array}$$ (3)

We assume that the riskier sector needs to offer a larger income to employees to compensate for the risks involved (e.g. job loss or the negative health effects of high stress environments). This, however, is not the case in many countries.

### Estimation of the Sigma parameter

We empirically estimate Eq (1) using data for Croatia [22], an EU member with a relatively high wage premium (22%) and relatively high corruption (CPI = 47) (see Fig 2). Taking into account several factors—the average tenure in both sectors, the relative risk of job loss, the intention of workers to change jobs, the percentage of workers in temporary positions, the average working hours, and the wage inequality (the percentage of workers with a wage below 60% of the medium wage)—we find that a job in the private sector is on average 2.5 to 3 times riskier than a job in the public sector. Thus *σ*_*pr*_ in Croatia has a value that is 2.5 to 3 times higher than *σ*_*pu*_. Nevertheless, the average wage for a lower-risk public job is 22% higher than the average wage for a higher-risk private job. Therefore, contrary to economic logic wages in the public sector, although less risky and providing greater benefits, are higher than wages in the private sector.

## Model

In the literature on corruption one of the central assumptions is that corruption is a function of motivation and opportunity [23] where, e.g., the rich are motivated to engage in corrupt activities as inequality in society increases. We expand this conflict between the rich and the poor to the public and private sectors. Here we assume that through corrupt activities public sector will attempt to maintain such privileges as larger salaries and lower sector risk.

The literature on models of corruption proposes that either corruption creates income inequalities [24] or the reverse [25]. According to Olson’s theory [26] corruption in the form of repression comes first and provides the underlying cause, or at least the motivation, for inequality. At the dawn of civilization, people living in small tribal communities were subject to the constant threat of roving bandits. In order to seek protection from the roving bandits the primitive forms of tribal societies established a *stationary bandit* to whom they paid taxes in exchange for protection from roving bandits. This was the initial motivation for the creation of the state—to have a *stationary bandit* to whom people pay taxes in exchange for protection from theft and expropriation. Thus a condition of “certain repression” replaced that of uncertain repression, and a class-based society was created out of which all future social orders have developed.

We do not try to determine historically and institutionally which came first, corruption or the gap (inequality) in incomes. We simply assume that a country has both a given wage gap and level of corruption (see Fig 1). We do however apply the same analogy and observe how countries characterized by different levels of income inequality can switch from a corrupt to a non-corrupt state.

Note that wage gaps and corruption could emerge together. If a less risky public sector job is paid more than a riskier private sector job, if the country is democratic and there is no corruption the system will not last. Note that Fig 1 reveals that for given wage gap every more corrupt country has as a counterpart in a less corrupt country. Is there a mechanism that causes a country to switch from a predominantly corrupt to a predominantly noncorrupt? How can a political and economic system that clearly defies economic logic be sustainable in the long run? To answer these questions, we next propose a non-linear network model that explains how a country that starts out more corrupt can stochastically switch to being less corrupt.

### Model For Two Phases

**Fig 4:**
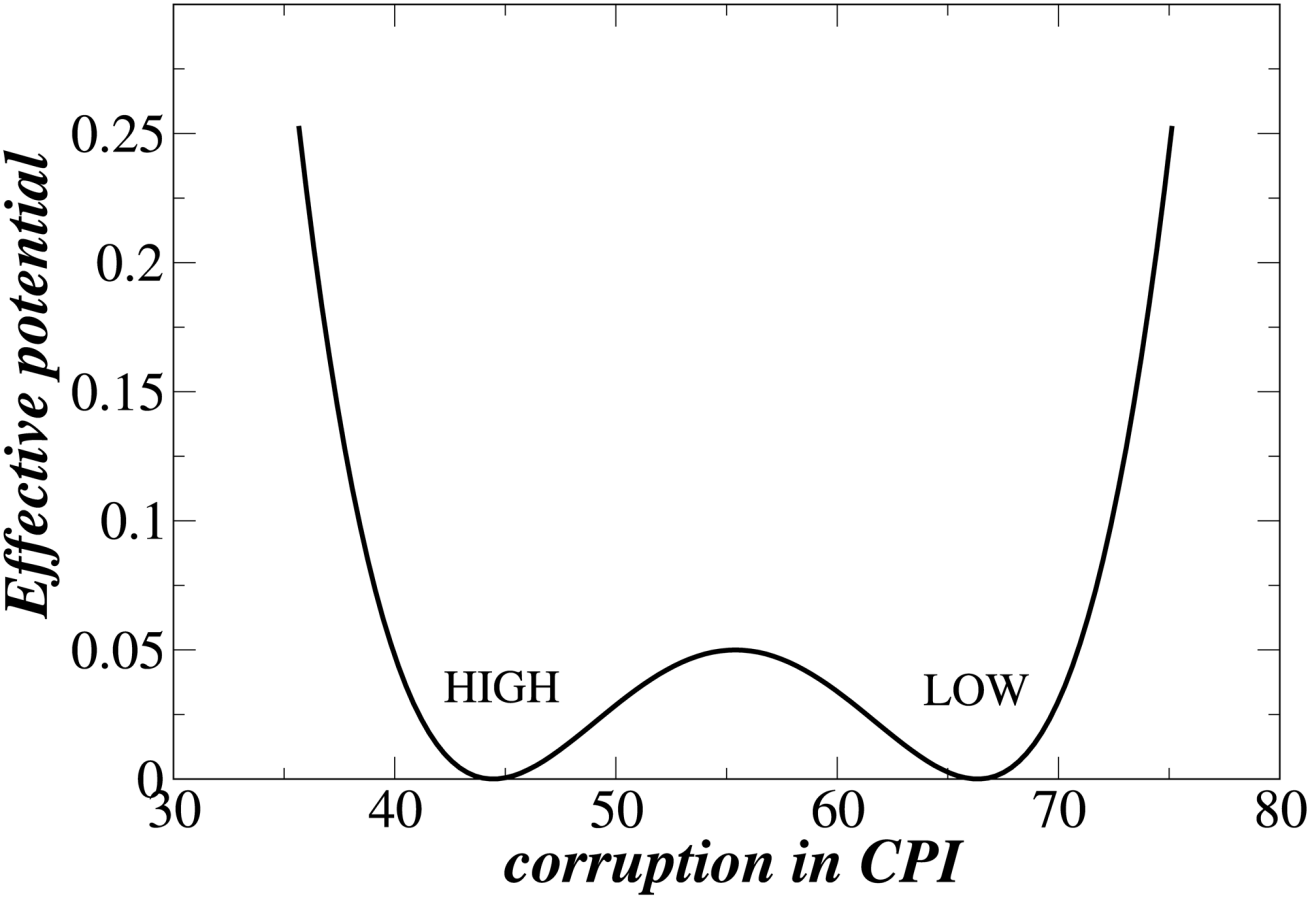
Illustrations of effective potentials. For a given wage premium two phases are shown corresponding to low and high corruption levels.

Using the results shown in Fig 2, we next determine the probability that a corrupt EU country, e.g., Greece, will substantially reduce its corruption to a lower level, e.g., that of Sweden. We hypothesize that, for a voting population, the two phases **I** and **II** correspond to two different levels of corruption for the same wage premium—in **I** the majority of citizens are prone to corruption and in **II** only a minority are prone to corruption. Fig 4 shows that phases **I** and **II** are two states in the nonlinear double-well potential between public and private sectors illustrated in Fig 1, where the potential barrier between these two states makes the transfer difficult. For example, when we put a system in the more corrupt state **I**, it slides to the bottom of the well and, if there are no fluctuations, it stays there. If there were no fluctuations of corruption in Greece, it would not have been able to move out of phase **I**. If the corruption is able to fluctuate, however, the system can jump over the potential barrier and move to phase **II**, and the larger the fluctuations, the larger the probability that the transfer can occur.

### Model with a fixed wage gap and democracy not included

The labor market in any country is composed of public and private sectors, each occupied by both corrupt and noncorrupt agents. In our model corruption is sustained not only by those who actively participate in corrupt activities, such as giving or taking bribes, but also by those members of the private sector who do business with the corrupt part of the public sector. The state of corruption is characterized by the fraction of society affected by corruption, and the larger the fraction, the greater the corruption. By the same rationale, the larger the wage gap, the more willing are the public sector employees to protect their rights and privileges. In the network model the labor force is characterized by its inherent corruption level. Hereafter we assume that the fraction *q* (1 − *q*) of all agents are inherently corrupt (noncorrupt) where, without loss of generality, half of the inherently corrupt agents are in the public sector, and their behavior is the same irrespective of the sector to which they belong. However each inherently noncorrupt (corrupt) agent can become corrupt (noncorrupt) if they are surrounded by a critically large fraction of corrupt (noncorrupt) agents [27].

**Fig 5:**
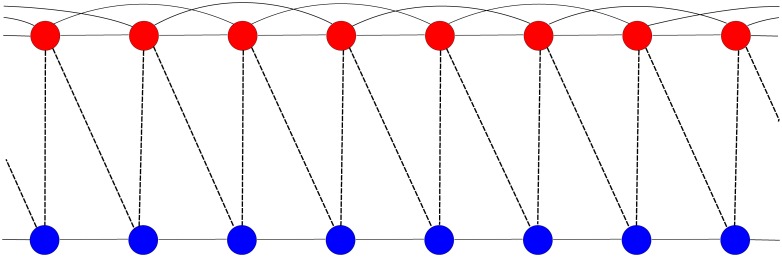
Two regular network models where corrupt agents (red) are more intra-linked than uncorrupt agents (blue). Here *k*_*c*_ = 4, *k*_*u*_ = 2, and *k*_*cu*_ = 2.

We define inherently corrupt agents as forming an Erdős-Renyi network ER I in which intra-network links are randomly chosen and their average number is *k*_*c*_. Similarly, inherently noncorrupt agents form another Erdős-Renyi network ER II in which the average number of intra-links is *k*_*u*_ < *k*_*c*_ (see Fig 5). We demonstrate below that the condition *k*_*c*_ > *k*_*u*_ is why inherently corrupt agents can dominate inherently noncorrupt agents, even when the latter group outnumbers the former. Once both networks are created, we randomly link agents from ER I with agents from ER II, where the average number of inter-network links is *k*_*u*, *c*_. Inter-network links serve to switch the attitude of the agents in the competing group.

In the network model corrupt agents in the public sector tend to be more interconnected than noncorrupt agents in order to keep the asymmetry in rights and incomes between the public and private sector in favor of the public sector. Here corrupt agents in the private sector do not benefit directly from the wage gap, but because they are connected to corrupt public sector agents, they can benefit from exclusive public procurement contracts or prone legislation.

As stated above, we allow each agent to switch their stance toward corruption in two ways, internally or externally [28]. Internally a corrupt agent from network ER I can, with a probability *p*_1,*c*_, become noncorrupt for a finite period of time *τ* (e.g., the time period between two elections). Similarly, an noncorrupt agent from network ER II can, with a probability *p*_1,*u*_, become corrupt during a period *τ*—in practice the media may affect these internal transitions. Externally, based on a threshold concept [27], each agent can also be influenced by their neighbors to change their opinion. If the total fraction of corrupt neighbors of a corrupt agent *i* is less than a fractional threshold *T*_*c*_ [29], with the probability *p*_2,*c*_ the corrupt agent *i* will become externally noncorrupt. Similarly, if the total fraction of noncorrupt neighbors of an noncorrupt agent *i* is less than a fractional threshold *T*_*c*_ [29], then the probability that an noncorrupt agent *i* will become externally corrupt is *p*_2,*u*_. Thus if the majority of local contacts are corrupt, remaining noncorrupt is a disadvantage. When a country is highly corrupt it is generally more likely that noncorrupt agents will become corrupt than vice versa, so *T*_*u*_ > *T*_*c*_. For reasons of simplicity, we hereafter set *T*_*u*_ = 1 − *T*_*c*_, where *T*_*c*_ is determined by the level of corruption. In numerical simulations for simplicity we set *p*_1,*c*_ = *p*_1,*u*_ ≡ *p*_1_ and *p*_2,*u*_ = *p*_2,*c*_ ≡ *p*_2_ even though the more appropriate would be *p*_1,*c*_ < *p*_1,*u*_, i.e., the noncorrupt agents more readily become corrupt than the reverse.

**Fig 6:**
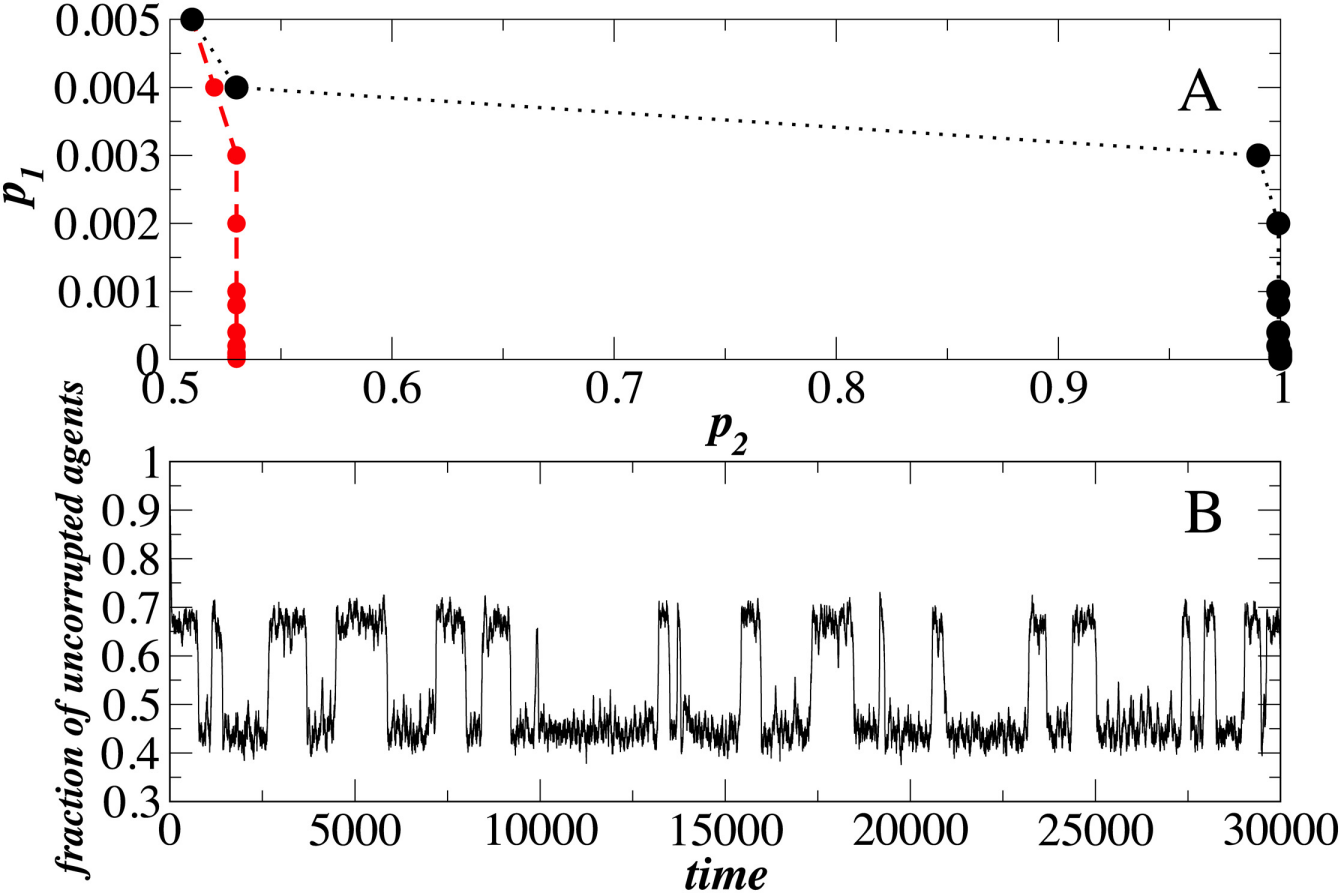
(A) As a result of competing between two networks we show the phase space of network II. We used *τ* = 50, *T_h_* = 0.5. (B) Close to a critical point, *p*1 = 0.004, *p*2 = 0.51, where *τ* = 50, *T_h_* = 0.5 we show the phase flipping between two phases. We use *q* = 0.25.

We next analyze network ER II. In order to ensure a voter majority, the inherently corrupt agents in ER I must successfully influence a fraction of noncorrupt agents in ER II to become corrupt. Fig 6A shows for network ER II the phase diagram in model parameters (*p*_1_, *p*_2_), where the hysteresis exhibits a first-order phase transition [28]. The hysteresis region is bounded by two spinodals merging at a critical point [28]. The right spinodal is obtained by increasing *p*_2_ starting from agents that are each externally and internally functional in both networks. The left spinodal is obtained by decreasing *p*_2_, starting from agents that are each internally functional but externally dysfunctional in both networks. When the model parameters are set at the critical point [see Fig 6A], Fig 6B shows how the fraction of noncorrupt agents in network ER II flips back and forth between two phases. When the fraction flips to a lower state the majority of agents in ER II become corrupt. Phase-flipping [28] is characteristic of a random walker put in an effective double-well potential such as the one in Fig 6. By phase-flipping behavior we mean a special property of dynamic networks, when for some choice of network parameters the network may spontaneously jump from one state to another and this process may hold repeatedly. Recently phase flipping has been modeled in finance [28] and economics [29]. In economics we may say that years of recession and years of expansion represent these phase-flipping states. In section Model For Two Phases we argue that in our model phase flipping occurs when corruption (here modeled by parameters *p*_1_ and *p*_2_) is allowed to fluctuate, which makes the system repeatedly jump over the potential barrier. However, the magnitude of fluctuations depends on the size of the system, and since the number of agents in public and private sectors is generally large, it is not likely that this phase-flipping phenomenon will occur among countries with different corruption levels.

**Fig 7:**
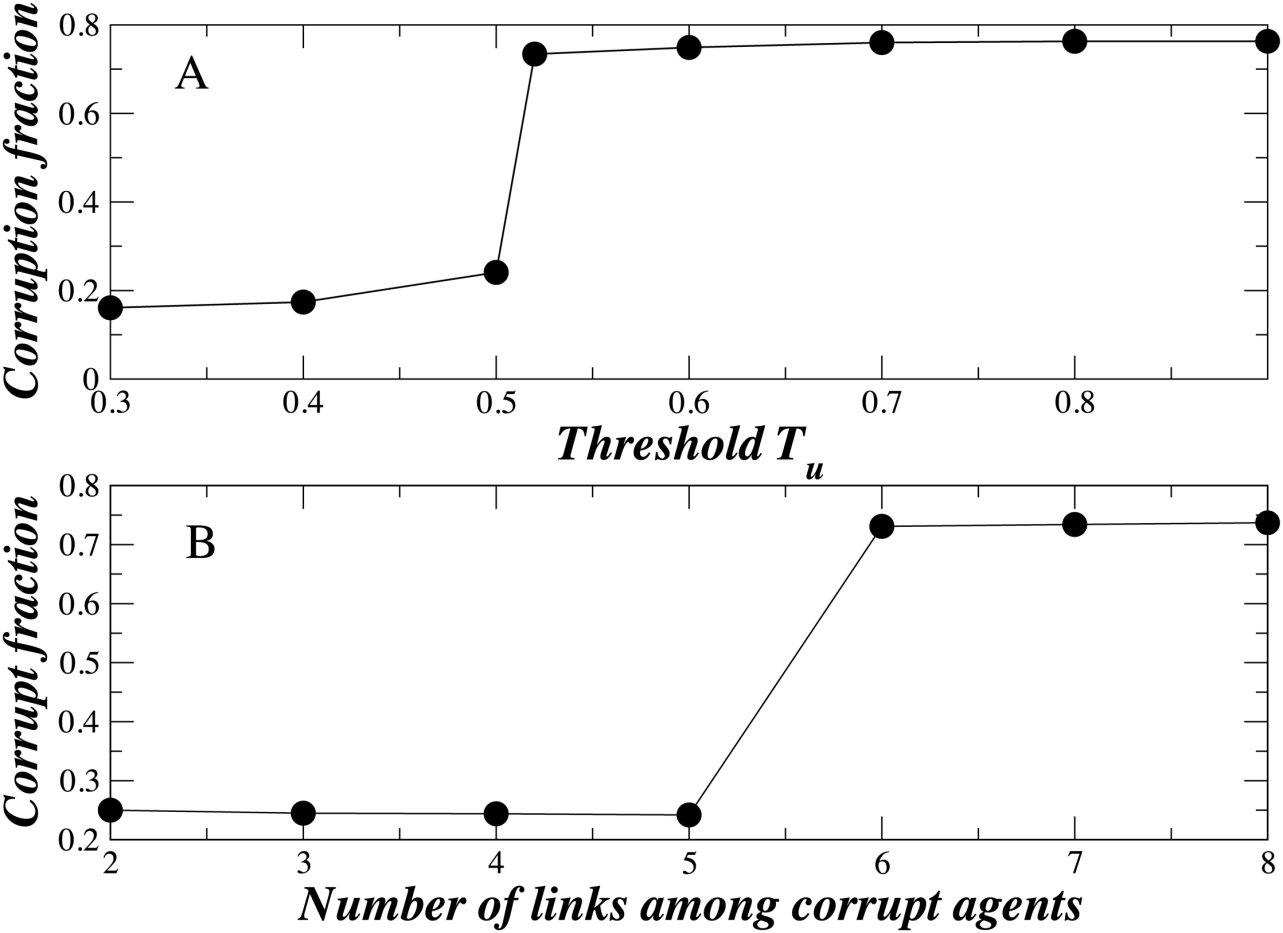
Fraction of corrupt agents increases with the number of intra-links and the threshold. (A) With increasing the vulnerability of noncorrupt agents in a corrupt surrounding, here quantified by threshold *T*_*u*_, the corrupt fraction increases. We use *p*_1_ = 0.002, *p*_2_ = 0.7, *k*_*u*_ = 2, *k*_*c*_ = 5, *k*_*uc*_ = 2. We use 2000 agents with 25% of corrupt agents. (B) With increasing the number of contacts established by corrupt agents, the corrupt fraction abruptly increases at one *k*_*c*_ value. We use *T*_*c*_ = 0.5, *k*_*u*_ = 2, and *k*_*uc*_ = 2.

In the network model the parameters *T*_*c*_, *k*_*c*_, *k*_*u*_, and *k*_*uc*_ control the level of corruption of a given country. Fig 7A shows, for a given set of parameters, how the majority of all agents in ER I and ER II suddenly become corrupt as the *T*_*c*_ value approaches 0.5, despite the initially larger fraction of inherently noncorrupt agents. Fig 7B shows that when the number of intra-links between corrupt agents is increased and the number of intra-links between noncorrupt agents is fixed the majority of agents abruptly become corrupt. This demonstrates how a minority of inherently corrupt agents can achieve an electoral majority—they make certain that the number of their intra-linked connections exceeds those of the noncorrupt agents. Note that the network formalism enables a discontinuous and sudden jump from one state to another, similar to switching processes between different regimes in stochastic processes (e.g. Markov Regime Switching Models) [30].

### Mean-field theory based on random graphs

Using mean-field theory based on random regular topology, we next analytically describe the interaction between corrupt and uncorrupt agents. In both networks, *d* ≡ 1 − *f* is the fraction of “disfunctional” agents, where *f* is the fraction of “functional” agents, differently defined in ER I and ER II. In network ER I corrupt agents are functional and the uncorrupt disfunctional. In network ER II the reverse is true. Assuming that internal and external failures are independent, we approximate the values of *d* in each network by

$$\begin{array}{ccc} d_{c}\equiv 1-f_{c} & = & p^{*}+p_{2}\left(1-p^{*}\right)E_{c}\\ d_{u}\equiv 1-f_{u} & = & p^{*}+p_{2}\left(1-p^{*}\right)E_{u}, \end{array}$$ (4)

where p* = exp(−*τp*_1_) is the average fraction of internally disfunctional agents [28] and *p*_2_ *E*_*S*_ the probability that a corrupt agent in network I with a critically damaged neighberhood quantified by *E*_*c*_ has externally become uncorrupt, where

$$\begin{array}{ccc} E_{c} & = & \Sigma_{j=0}^{t_{c}}\Sigma_{i=0}^{j}\left(\begin{array}{c} k_{c}\\ k_{c}-i \end{array}\right)d_{c}^{k_{c}-i}f_{c}^{i}\left(\begin{array}{c} k_{u,c}\\ k_{u,c}-\left(j-i\right) \end{array}\right)f_{u}^{k_{u,c}-\left(j-i\right)}d_{u}^{j-i}\\ E_{u} & = & \Sigma_{j=0}^{t_{u}}\Sigma_{i=0}^{j}\left(\begin{array}{c} k_{u}\\ k_{u}-i \end{array}\right)d_{u}^{k_{u}-i}f_{u}^{i}\left(\begin{array}{c} k_{c,u}\\ k_{c,u}-\left(j-i\right) \end{array}\right)f_{c}^{k_{c,u}-\left(j-i\right)}d_{c}^{j-i}. \end{array}$$ (5)

Here *t*_*c*_ represents the absolute threshold of network ER I—a corrupt agent in ER I can externally fail and become uncorrupt with a probability *p*_*c*,2_ only when the number of uncorrupt neighbors in both network ER I and network ER II is lower than or equal to *t*_*c*_. We obtain *E*_*u*_ for network ER II from *E*_*c*_ when *u* ⇔ *c*.

Recalling that *q* denotes the fraction of inherently corrupt agents, we find that the fraction of corrupt agents is

$$\begin{array}{c} F_{tot}=q\left(1-d_{c}\right)+\left(1-q\right)d_{u}. \end{array}$$ (6)

Here *d*_*u*_ and *d*_*c*_ are defined as in Eq (4). For a given *p*_1_ and *p*_2_, we can find sets of parameters (*k*_*c*_, *k*_*u*_, *k*_*u*, *c*_) for which the fraction of corrupt agents becomes a majority, $F_{\text{tot}}>0.5$, $F_{\text{tot}}>0.5$ can be achieved by keeping *d*_*c*_ as small as possible and *d*_*u*_ as large as possible [see Fig 4B]. Note that from Eqs (4)–(6), as *t*_*c*_ becomes smaller, the *E*_*c*_ does as well, and this implies a smaller *d*_*c*_. Similarly, the larger the *t*_*u*_ value, the larger the *E*_*u*_ value, and this implies a larger *d*_*u*_.

Note that, due to finite-size effects and stochasticity, in relatively small samples the local-time sample realizations in *p*_1_(*t*) and *p*_2_(*t*) can substantially fluctuate from the population values [28] *p*_1_ and *p*_2_. This causes a spontaneous and dramatic switch between ER I and ER II. In a very large sample, e.g., the entire population of a country, this corrupt-to-uncorrupt switch is not as probable [29].

### Model with variable wage gap where democracy is a mechanism of corruption preservation

The current literature reports that the relationship between democracy and corruption is inconclusive [31, 32]. We now add democracy to the network model for developing and developed countries described in Sec. 5, and exclude the leftmost part of the concave dependence shown in Fig 1.

We assume that when the members of a country’s parliament are randomly chosen from the country’s citizens, the random nature of the electoral system can result in a parliamentary membership that is more corrupt than the average corruption level of society. We also assume that the difference between the corruption level of the parliament and the average corruption level of society, *D*, proportionally changes the parameters controlling the corruption level (e.g., *k*_*c*_ and *T*_*c*_) and increases both the corruption level and the wage gap. In general, the larger the fluctuations in *D*, the larger the probability that a switch from a predominantly corrupt (**I**) to a predominantly noncorrupt (**II**) phase will occur. On the other hand, when parliament members are less corrupt than the average corruption level of the society, the parameters change, i.e., the threshold *T*_*u*_ is decreased, the country is less corrupt, and the wage gap is reduced. Thus if the majority of citizens of a democratic country are prone to corruption, it is probable that the country’s parliamentary membership—which is a randomly chosen sample—will reflect that same level of corruption. Thus, according to our model, democracy is not responsible for the emergence of corruption, but can be responsible for its persistence. This theoretical result is in agreement with empirical studies that indicate that lowering corruption in a democracy requires a period of time in excess of forty years [9].

We see an inherent flaw of democracy in such corrupt systems. According to the logic of the selectorate theory [33], in democracies characterized by a set of extractive economic and political institutions [34] the political elites design a system that supports their rent-extracting activities. They are able to stay in power by maintaining a *winning coalition* of supporters, and they do this by building and maintaining a legitimate electoral majority. Because the political structure is democratic, they do this by supplying their supporters with public goods.

Thus, in our model, democracy acts as a stabilization mechanism for the system, irrespective of corruption level. In corrupt democratic societies the corrupt electoral majority is highly intra-linked, the legislative body faithfully represents them, and the corruption level is sustained. In societies with a low level of corruption the mechanism is the same: the electoral majority is not corrupt, the societal network is homogeneously interconnected, and the democratic mechanisms isolate the corrupt minority and minimize its influence on the government and its institutions.

It follows that in a system in which the majority of participating voters are corrupt, we will find a Nash equilibrium in which democracy sustains corruption. The opposite is true for societies in which the majority of voters are noncorrupt. Thus in a corrupt society we will find parameter sets *k*_*c*_, *k*_*u*_, *k*_*u*, *c*_ (see Eq (5)) for which the majority of agents who vote will engage in corrupt practices ($F_{tot}>0.5$). At higher levels of corruption we assume these agents gain an increasing amount of influence. When this group of interconnected corrupt voters achieve an electoral majority (Fig 5) their optimal response strategy is to choose politicians who will allow them to continue their corrupt practices. For a Nash equilibrium to hold, none of the agents can have an incentive to deviate. If they were to deviate and vote for congruent (non-corrupt) politicians, their ability to influence the government would be reduced. This Nash equilibrium is self-reinforcing as long as the corrupt outnumber the uncorrupt. Democracy thus does not create corruption, but it can be used as a tool for sustaining it when the electoral majority is corrupt.

Here we disregarded the role of political parties and how they maintain the equilibrium, particularly when both parties are equally corrupt. If agents choose their parties randomly, then the distribution of members in a party will reflect the distribution of the electorate. This means that the statistics for large samples will be very similar to the statistics for the entire population. In other words, in a corrupt society all political parties will have more corrupt agents than noncorrupt.

Because all parties compete for the same electorate, a change in the ruling political party will not necessarily change the country’s level of corruption. Thus a flip from a corrupt to a non-corrupt state can only occur when random selection happens to place a non-corrupt person in the position of head of state.

For example, in Croatia both two main political parties (and some of their coalition partners) became engulfed in corruption scandals, having their high-ranking party officials ending up in prison on corruption charges. Similar scenarios can be observed in many other countries that suffer from the same structural wage imbalance. For example in Italy, after decades of rule by the Christian Democrats their corrupt practices were finally exposed in 1992 under the *Tangentopoli* affair. Although this nation-wide investigation effectively destroyed the Christian Democrats, it also led to charges against the main opposition party, the Social Democrats and its leader Bettino Craxi, who became a symbol of political corruption in Italy.

## Conclusion

In financial markets stocks are riskier than bonds because they promise a higher return. Were the opposite true and low-risk investments generated higher returns, the distorted investment signals would collapse the market. This is not the case in labor economics, where low-risk public sector jobs are often better paid than high-risk private sector jobs. We examine this wage gap paradox for 28 EU member states and find that those with a larger wage gap have a higher level of corruption than those with a lower wage gap. We define a new reward-to-risk labor ratio to compensate for varying risks of different sectors. We propose a dynamical inter-connected network model in which inherently corrupt and noncorrupt agents compete to achieve dominance, and where democracy serves as a mechanism that preserves corruption.
